# Protecting the gains: analysis of HIV treatment and service delivery programme data and interventions implemented in 19 African countries during COVID‐19

**DOI:** 10.1002/jia2.26033

**Published:** 2022-11-23

**Authors:** Pamela J. Bachanas, Helen M. Chun, Neha Mehta, John Aberle‐Grasse, KaeAnne Parris, Michelle Williams Sherlock, Spencer Lloyd, Clement Zeh, Daphne K. Makwepa, Max L. Kapanda, Emily Kainne Dokubo, Leonard Bonono, Shirish Balachandra, Eboi Ehui, Peter Fonjungo, Aimé M. Nkoso, Sikhathele Mazibuko, Velephi N. Okello, Fana Tefera, Mirtie Getachew, Elizabeth M. Katiku, Andrew Mulwa, Fred M. Asiimwe, Tapiwa F. Tarumbiswa, Andrew F. Auld, Rose Nyirenda, Alzira P. Dos Santos De Louvado, Irenio Gaspar, Steven Y. Hong, Laimi Ashipala, Christopher Obanubi, Akudo Ikpeazu, Canisious Musoni, Emmanuel Yoboka, Simangele Mthethwa, Zukiswa Pinini, Sudhir Bunga, John Rumunu, Daniel J. Magesa, Beatrice Mutayoba, Lisa J. Nelson, Cordelia Katureebe, Simon Agolory, Lloyd B. Mulenga, Ponesai Nyika, Owen Mugurungi, Tedd Ellerbrock, Kiren Mitruka

**Affiliations:** ^1^ Division of Global HIV/AIDS and TB Centers for Disease Control and Prevention Atlanta Georgia USA; ^2^ Division of Global HIV/AIDS and TB Centers for Disease Control and Prevention Gaborone Botswana; ^3^ National ART Program Ministry of Health and Wellness Gaborone Botswana; ^4^ Division of Global HIV/AIDS and TB Centers for Disease Control and Prevention Yaounde Cameroon; ^5^ National AIDS Control Committee Ministry of Public Health Yaounde Cameroon; ^6^ Division of Global HIV/AIDS and TB Centers for Disease Control and Prevention Abidjan Cote d’ Ivoire; ^7^ National AIDS Control Program Ministry of Health and Public Hygiene Abidjan Cote d’ Ivoire; ^8^ Division of Global HIV/AIDS and TB Centers for Disease Control and Prevention Kinshasa Democratic Republic of the Congo; ^9^ Ministry of Health Kinshasa Democratic Republic of Congo; ^10^ Division of Global HIV/AIDS and TB Centers for Disease Control and Prevention Mbabane Eswatini; ^11^ Health Services – Clinical Ministry of Health Mbabane Eswatini; ^12^ Division of Global HIV/AIDS and TB Centers for Disease Control and Prevention Addis Ababa Ethiopia; ^13^ Disease Prevention and Control Directorate Ministry of Health Addis Ababa Ethiopia; ^14^ Division of Global HIV/AIDS and TB Centers for Disease Control and Prevention Nairobi Kenya; ^15^ Preventive and Promotive Health Ministry of Health Nairobi Kenya; ^16^ Division of Global HIV/AIDS and TB Centers for Disease Control and Prevention Maseru Lesotho; ^17^ Ministry of Health Maseru Lesotho; ^18^ Division of Global HIV/AIDS and TB Centers for Disease Control and Prevention Lilongwe Malawi; ^19^ Ministry of Health Lilongwe Malawi; ^20^ Division of Global HIV/AIDS and TB Centers for Disease Control and Prevention Maputo Mozambique; ^21^ HIV Program Ministry of Health Maputo Mozambique; ^22^ Division of Global HIV/AIDS and TB Centers for Disease Control and Prevention Windhoek Namibia; ^23^ HIV/STI Sub‐Division, Directorate Special Programmes Ministry of Health and Social Services Windhoek Namibia; ^24^ Division of Global HIV/AIDS and TB Centers for Disease Control and Prevention Abuja Nigeria; ^25^ National AIDS/STI Control Programme Federal Ministry of Health Abuja Nigeria; ^26^ Division of Global HIV/AIDS and TB Centers for Disease Control and Prevention Kigali Rwanda; ^27^ Ministry of Health Kigali Rwanda; ^28^ Division of Global HIV/AIDS and TB Centers for Disease Control and Prevention Pretoria South Africa; ^29^ HIV, AIDS & STIs Cluster National Department of Health Pretoria South Africa; ^30^ Division of Global HIV/AIDS and TB Centers for Disease Control and Prevention Juba South Sudan; ^31^ Preventive Health Services Ministry of Health Juba Republic South Sudan; ^32^ Division of Global HIV/AIDS and TB Centers for Disease Control and Prevention Dar es Salaam Tanzania; ^33^ Ministry of Health Dar es Salaam Tanzania; ^34^ Division of Global HIV/AIDS and TB Centers for Disease Control and Prevention Kampala Uganda; ^35^ Ministry of Health Kampala Uganda; ^36^ Division of Global HIV/AIDS and TB Centers for Disease Control and Prevention Lusaka Zambia; ^37^ Ministry of Health Lusaka Zambia; ^38^ Division of Global HIV/AIDS and TB Centers for Disease Control and Prevention Harare Zimbabwe; ^39^ Ministry of Health Harare Zimbabwe

**Keywords:** COVID‐19, treatment, viral suppression, retention, treatment interruption, differentiated care

## Abstract

**Introduction:**

The potential disruption in antiretroviral therapy (ART) services in Africa at the start of the COVID‐19 pandemic raised concern for increased morbidity and mortality among people living with HIV (PLHIV). We describe HIV treatment trends before and during the pandemic and interventions implemented to mitigate COVID‐19 impact among countries supported by the US Centers for Disease Control and Prevention (CDC) through the President's Emergency Plan for AIDS Relief (PEPFAR).

**Methods:**

We analysed quantitative and qualitative data reported by 10,387 PEPFAR‐CDC‐supported ART sites in 19 African countries between October 2019 and March 2021. Trends in PLHIV on ART, new ART initiations and treatment interruptions were assessed. Viral load coverage (testing of eligible PLHIV) and viral suppression were calculated at select time points. Qualitative data were analysed to summarize facility‐ and community‐based interventions implemented to mitigate COVID‐19.

**Results:**

The total number of PLHIV on ART increased quarterly from October 2019 (*n* = 7,540,592) to March 2021 (*n* = 8,513,572). The adult population (≥15 years) on ART increased by 14.0% (7,005,959–7,983,793), while the paediatric population (<15 years) on ART declined by 2.6% (333,178–324,441). However, the number of new ART initiations dropped between March 2020 and June 2020 by 23.4% for adults and 26.1% for children, with more rapid recovery in adults than children from September 2020 onwards. Viral load coverage increased slightly from April 2020 to March 2021 (75–78%) and viral load suppression increased from October 2019 to March 2021 (91–94%) among adults and children combined. The most reported interventions included multi‐month dispensing (MMD) of ART, community service delivery expansion, and technology and virtual platforms use for client engagement and site‐level monitoring. MMD of ≥3 months increased from 52% in October 2019 to 78% of PLHIV ≥ age 15 on ART in March 2021.

**Conclusions:**

With an overall increase in the number of people on ART, HIV programmes proved to be resilient, mitigating the impact of COVID‐19. However, the decline in the number of children on ART warrants urgent investigation and interventions to prevent further losses experienced during the COVID‐19 pandemic and future public health emergencies.

## 1 INTRODUCTION

As of 10 March 2022, the World Health Organization (WHO) reported 8,134,850 confirmed COVID‐19 cases and 169,676 deaths in the WHO Africa region, representing 1.8% of cases and 2.8% of deaths worldwide [1]. While the early waves of COVID‐19 resulted in high numbers of cases and deaths, the reported magnitude of the pandemic has been comparably less in Africa than most other continents. Nevertheless, the potential for devastating COVID‐19‐related morbidity and mortality has remained a concern due to limited resources, overstretched health systems, and the high prevalence of HIV and other infectious diseases [2, 3]. Poorer health outcomes have been demonstrated with the co‐infection of HIV and SARS‐CoV‐2, the virus that causes COVID‐19 [4, 5, 6]. Of the 38 million people living with HIV (PLHIV) worldwide, approximately 25.6 million reside in Africa [7]. Modelling data estimated 6‐month HIV treatment interruptions could have resulted in over 400,000 additional deaths in African countries in 2020–2021 [8]. Therefore, to protect the hard‐won gains towards the UNAIDS targets of ending the HIV epidemic by 2030 [9], continued vigilance is needed to fight the COVID‐19 pandemic in Africa.

The President's Emergency Plan for AIDS Relief (PEPFAR) supports over 25 countries in Africa to achieve HIV epidemic control [10]. The US Centers for Disease Control and Prevention (CDC) is one of PEPFAR's key agencies that collaborates with Ministries of Health (MOHs) to support national HIV programmes.

Prior to COVID‐19, most PEPFAR‐supported countries were implementing person‐centred, differentiated antiretroviral therapy (ART) service delivery models [11, 12, 13]. Many programmes had implemented multi‐month dispensing (MMD) (3 months or more) of antiretrovirals (ARVs) with reduced clinical visits (e.g. every 6–12 months), adherence clubs, fast‐track pharmacy refills and community ART pick‐up [14, 15, 16] with reported improved retention, and viral suppression (VS) [17, 18, 19].

Although the timing of the waves of COVID‐19 cases varied among countries, most countries in southern and eastern Africa experienced three waves with peaks between June—August 2020, December 2020–March 2021 and June—August 2021 [1]. HIV service disruptions were attributed to movement restrictions, facility shutdowns, clinic and HIV PCR laboratories’ repurposing, healthcare worker reallocation, health facility avoidance due to fears of contracting SARS‐CoV‐2 and healthcare worker infections [20, 21]. In coordination with MOHs, PEPFAR guided programmes towards four main priorities: continuity of care, reducing staff and client exposure to COVID‐19, leveraging PEPFAR‐supported health platforms to mitigate the impact of COVID‐19 and flexible person‐centred service delivery [22]. CDC worked with MOHs and partners, including community‐based organizations, to scale up and innovate interventions along these four priorities to protect the gains of HIV programmes [23, 24].

We describe HIV treatment trends prior to and during the COVID‐19 pandemic, and interventions implemented by PEPFAR‐CDC‐supported ART sites in 19 African countries to mitigate its negative impact.

## 2 METHODS

### 2.1 Study design and population

We analysed quantitative and qualitative routine HIV care and treatment programme data from PEPFAR‐CDC‐supported ART sites in 19 African countries (Botswana, Cameroon, Cote d'Ivoire, Democratic Republic of Congo [DRC], Eswatini, Ethiopia, Kenya, Lesotho, Malawi, Mozambique, Namibia, Nigeria, Rwanda, South Africa, South Sudan, Tanzania, Uganda, Zambia and Zimbabwe) which reported data to the Office of Global AIDS Coordinator (OGAC) between October 2019 and March 2021, with at least the first (October–December 2019) and last quarters (January–March 2021) reported. Sites that transitioned in or out of PEPFAR support or to another PEPFAR implementing agency during the first or last reporting period were excluded.

### 2.2 Data sources and measures

PEPFAR‐supported ART sites report aggregate, de‐identified data on client HIV services at least quarterly on Monitoring, Evaluation and Reporting (MER) indicators that span the HIV care cascade through a secured data system [25]. MER data are quarterly cross‐sectional snapshots rather than longitudinal assessments of clients in care over time. Data quality checks are performed for internal consistency and completeness at multiple levels, by ART sites, CDC HIV country programmes and OGAC before being finalized and included in structured datasets.

We analysed MER quarterly datasets for standard indicators of PLHIV currently on ART, new ART initiations, interruptions in treatment (IIT), viral load (VL) testing among eligible PLHIV on ART (i.e. viral load coverage [VLC]), VS and MMD of ARVs [26]. Currently on ART and new ART initiations were defined as the number of PLHIV on ART and newly initiating ART, respectively, during the reporting quarter. IIT was calculated as the number of PLHIV on ART who missed a scheduled appointment for an ART refill or clinical visit for ≥28 days divided by the number of PLHIV on ART in the preceding quarter plus the number that newly initiated ART in the reporting quarter. VLC was calculated as the number of PLHIV on ART who had a documented VL within 12 months of the reporting period divided by the number of PLHIV on ART 6 months prior to the period being analysed. VS was calculated as the number of PLHIV on ART who had a VL <1000 copies/ml divided by the number of PLHIV on ART with a documented VL in the past 12 months. The percentage of PLHIV who received MMD was calculated as the number of PLHIV on ART with an ARV supply for 3–5 months or ≥6 months divided by the number of PLHIV on ART during the reporting period.

Qualitative narratives and presentations on quarterly performance and COVID‐19 updates submitted by the 19 country HIV programmes were reviewed to identify themes in adaptations to service delivery due to COVID‐19. In addition, CDC programme staff from five countries (i.e. highlighted countries: Cameroon, DRC, Mozambique, Nigeria and Tanzania) that had ≥ 500,000 PLHIV and demonstrated the largest net increase in the number of PLHIV (adults and children) on ART at the end of the analytic period, January–March 2021, responded to a series of standardized questions and provided examples their country programmes implemented to mitigate the impact of COVID‐19.

### 2.3 Data analyses

We assessed quarterly trends in the number of PLHIV on ART, newly initiating ART and on MMD for the 19 countries combined. Based on the availability of denominator data, IIT trends were analysed from 1 January 2020 to 31 March 2021 and disaggregated by PLHIV on ART for <3 and ≥3 months. VLC was assessed for the period 1 April 2020–31 March 2021 as the denominator data (the number of PLHIV on ART eligible for VL testing) were not available before April 2020. VS was calculated for the first and last quarters of the analytic period. All analyses were stratified by adult (aged ≥15 years) and paediatric (aged <15 years) populations. To discern trends in people newly or currently on ART and increases in VLC and VS more granularly, analyses for these measures were also conducted for each of the five highlighted countries with the highest net increase in PLHIV currently on ART along with the examples of implemented interventions.

Descriptive statistics, including frequencies and quarterly percent change over time for currently and new on ART and percentage and percentage‐point change over time for IIT, VLC and VS, were calculated using Excel® 2016.

For the analysis of qualitative data, we created categories of common interventions based on recurring themes among the 19 countries, and systematically abstracted their interventions under each category and provided examples from the five highlighted countries.

### 2.4 Ethics

All data reported in this analysis were de‐identified, aggregate data obtained from regular reporting by PEPFAR. There were no perceived ethical risks to care recipients, therefore, no informed consent was obtained. This activity was conducted under a CDC protocol consistent with applicable federal law and CDC policy.

## 3 RESULTS

A total of 10,387 ART sites that reported data to the OGAC during both the first (October–December 2019) and last quarters (January–March 2021) of the analytic period were included in the analysis (4422 sites that did not report data in both the first and last quarters, including 2255 sites that transitioned to a PEPFAR implementing agency other than CDC were excluded).

### 3.1 Trends in treatment measures (19 countries)

During the 18‐month analytic period (October 2019–March 2021), the total number of PLHIV (including those with unknown age) currently on ART increased by 12.9% (from 7,540,592 to 8,513,572) in the 19 countries combined.

### 3.2 Adults (age 15 years and above)

**Figure 1:**
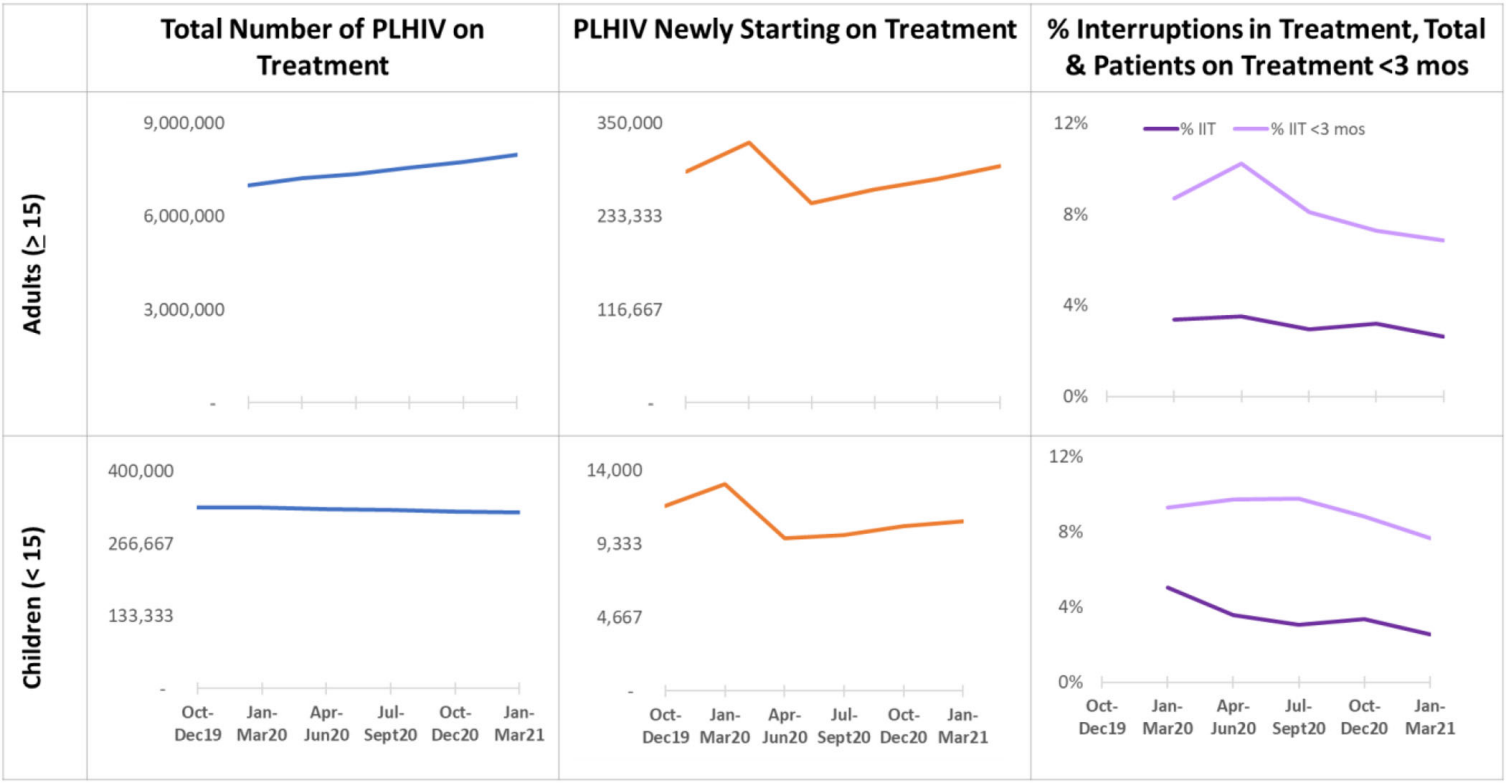
Trends in the total number of people living with HIV (PLHIV) on treatment, number of newly initiated PLHIV on treatment and the proportion of interruptions in treatment for adults (≥15) and children (<15) across 19 Centers for Disease Control‐President's Emergency Program for AIDS Relief‐Supported African Countries from October 2019 to March 2021.

Among PLHIV ≥15 years, the number currently on ART increased quarterly, overall, by 14.0% (from 7,005,959 to 7,983,793) during the analytic period (Figure 1). The number of new ART initiations dropped by 23.4% (from 326,252 to 250,061) from January to March 2020 to April to June 2020 but recovered to 297,029 in the following quarters of January to March 2021. The percentage of PLHIV with IIT (% IIT) fluctuated quarterly during January 2020–March 2021 with increases during April–June 2020 and October–December 2020. However, % IIT overall declined from 3.4% to 2.6% between January–March 2020 and January–March 2021. The % IIT for PLHIV on ART <3 months (% IIT <3 months) saw a sharp increase (8.7–10.2%) from January to March 2020 to April to June 2020 but declined steadily thereafter.

### 3.3 Children and adolescents (under age 15 years)

Among children living with HIV (CLHIV) <15 years of age, the number currently on ART decreased by 2.6% (from 333,178 to 324,441) in the 19 countries combined (Figure 1) during the analytic period. New ART initiations decreased by 26.1% (13,129–9707) from January to March 2020 to April to June 2020 with recovery thereafter up to 10,772 in January–March 2021. The % IIT across the analytic period ranged from 5.0% to 2.6%, with a decline each quarter until October–December 2020 when it increased by 9.0% and thereafter declined again. The % IIT<3 months ranged from 9.8% to 7.7% and increased quarterly until July–September 2020, and thereafter declined.

### 3.4 Trends in treatment measures in Cameroon, DRC, Mozambique, Nigeria and Tanzania

The five highlighted countries accounted for 4118 sites and 37% of the total PLHIV on ART included in the analysis during January–March 2021.

### 3.5 Adults (age 15 years and above)

**Figure 2:**
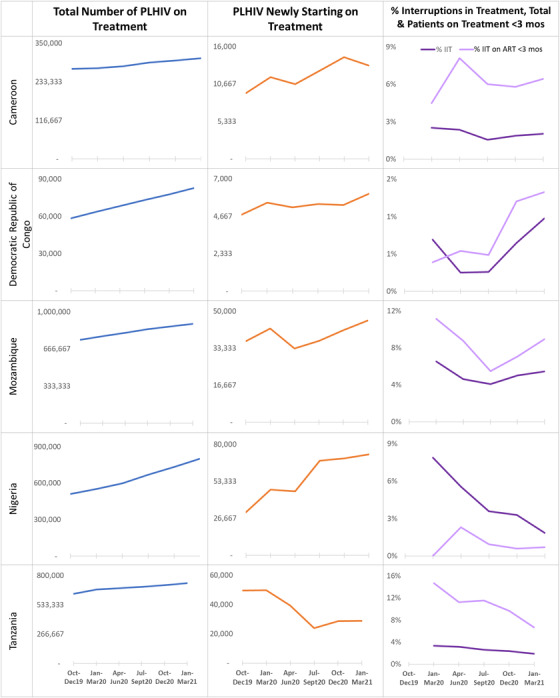
Trends in the total number of people living with HIV (PLHIV) on treatment, number of newly initiated PLHIV on treatment and the proportion of interruptions in treatment for adults (≥15) from October 2019 to March 2021 across five highlighted Centers for Disease Control‐President's Emergency Program for AIDS Relief‐Supported Countries.

Among PLHIV ≥15 years, the number currently on ART increased quarterly in each of the five countries by 11.7–57.1% (Figure 2). Four out of five countries observed an overall increasing trend in new ART initiations after an initial decline during April–June 2020 and recovered to above pre‐pandemic levels of October–December 2019 (overall net changes: Cameroon 42%, DRC 27%, Mozambique 26%, Nigeria 135% and Tanzania –42%). The % IIT and the % IIT <3 months followed varying trends in the different countries. While the % IIT improved to pre‐pandemic or lower rates in four of the five countries, the % IIT <3 months did not return to pre‐pandemic levels in three countries.

### 3.6 Children and adolescents (under age 15 years)

**Figure 3:**
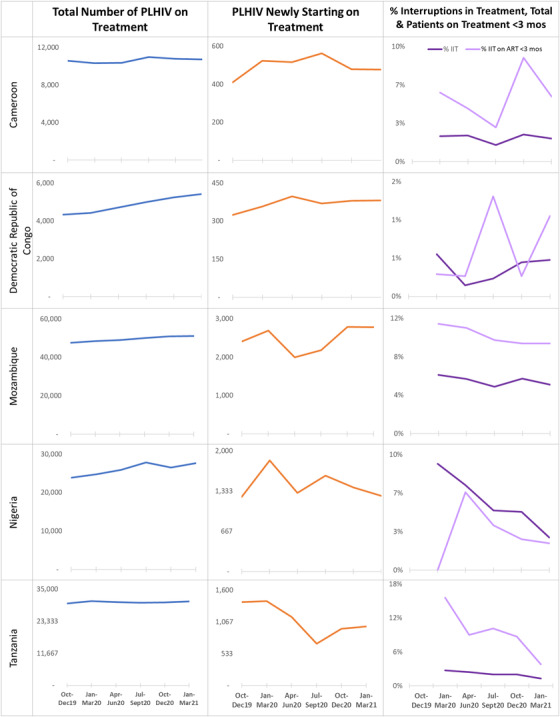
Trends in the total number of children living with HIV (CLHIV) on treatment, number of newly initiated CLHIV on treatment and the proportion of interruptions in treatment for chidren and adolescents (<15) from October 2019 to March 2021 across five highlighted Centers for Disease Control‐President's Emergency Program for AIDS Relief‐Supported Countries.

For each of the five highlighted countries, the number of CLHIV <15 years currently on ART increased quarterly by 1.2–25.1% (Figure 3). Countries observed fluctuations in the number of new ART initiations, with recovery to pre‐pandemic levels in four out of five countries during January–March 2021 (Figure 3). The % IIT and the % IIT <3 months also followed varying trends; while the % IIT improved to pre‐pandemic or lower rates in all five countries, the % IIT <3 months did not return to pre‐pandemic levels in the two countries during January–March 2021.

### 3.7 Trends in VL measures

In the 19 countries combined, the number of PLHIV (including CLHIV) with a documented VL increased from over 5.7 million during April–June 2020 to over 6.3 million during January–March 2021, and VLC increased by 3 percentage points between the same periods (Table 1). VS also improved by 3 percentage points among adults and 9 percentage points among children in the 19 countries combined between October–December 2019 and January–March 2021 (Table 1). In all five highlighted countries, VLC improved among both adults (range 1–20 percentage points) and children (range 3–11 percentage points) and VS improved among both adults (by 3–11 percentage points) and children (by 8–20 percentage points).

**Table 1:** Viral load coverage and viral load suppression among adults (≥15) and children (<15) across 19 Centers for Disease Control‐President's Emergency Plan for AIDS Relief‐Supported African Countries over time periods between October 2019 and March 2021

	Adults (> 15 years)				Children (<15 years)			
	Viral load coverage		Viral load suppression		Viral load coverage		Viral load suppression	
	Apr–Jun 2020	Jan–Mar 2021	Oct–Dec 2019	Jan–Mar 2021	Apr–Jun 2020	Jan–Mar 2021	Oct–Dec 2019	Jan–Mar 2021
19 countries	77%	79%	92%	95%	79%	81%	73%	82%
Cameroon	43%	63%	79%	90%	49%	60%	59%	70%
Dem Rep Congo	78%	79%	88%	93%	75%	78%	79%	87%
Mozambique	64%	68%	84%	92%	64%	71%	51%	71%
Nigeria	84%	88%	87%	94%	80%	83%	65%	81%
Tanzania	79%	83%	94%	97%	79%	82%	76%	89%

### 3.8 Interventions to mitigate the impact of COVID‐19

**Figure 4:**
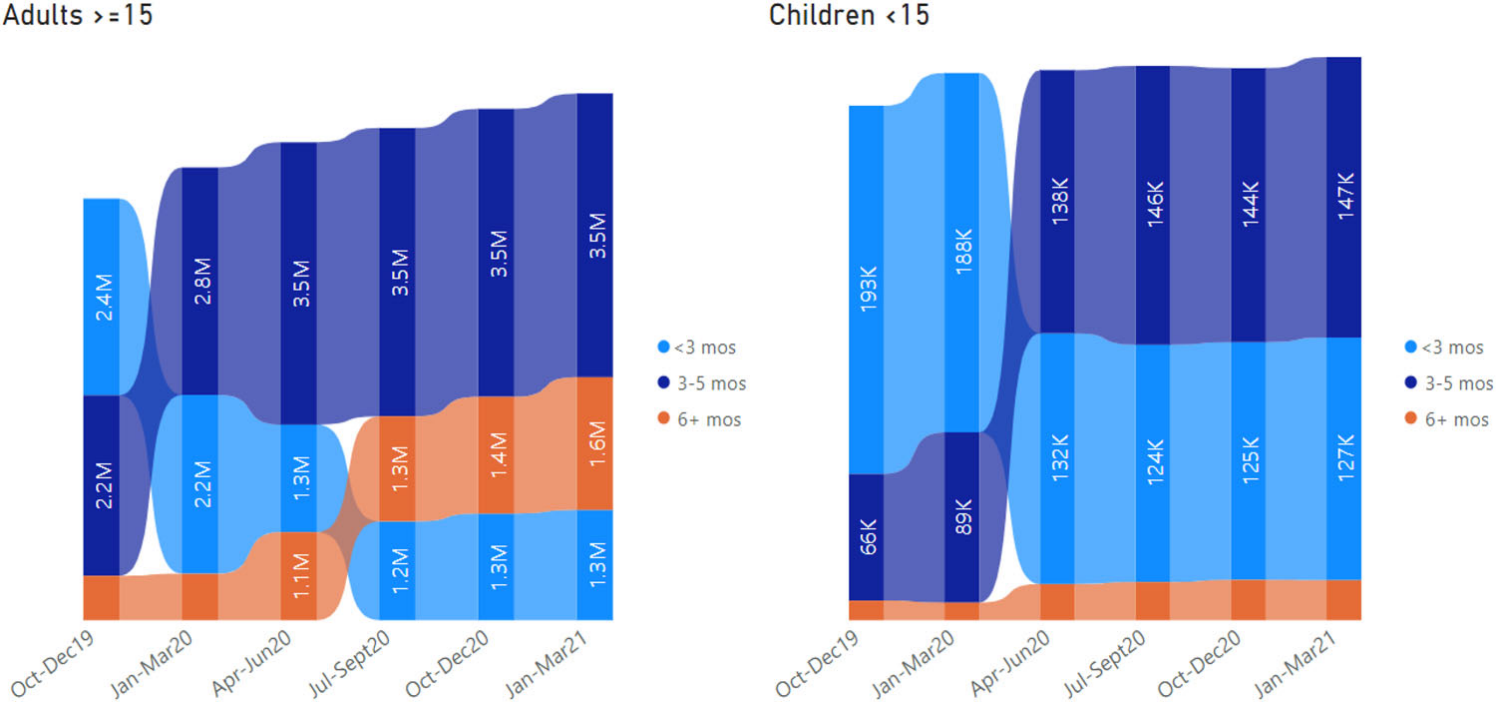
Trends in the scale up of multi‐month dispensing of ART 3–5 months and 6 months or more among adults (≥15 years) and children (<15 years) across 19 Centers for Disease Control‐President's Emergency Program for AIDS Relief‐Supported African Countries from October 2019 to March 2021.

In the 19 countries, the total number of PLHIV who received MMD ≥3 months nearly doubled (from 2,830,329 to 5,265,850) between October–December 2019 and January–March 2021 (Figure 4). The number of PLHIV aged ≥15 years on ART receiving MMD 3–5 months and ≥6 months increased from 2,210,606 to 3,473,785 (42–54% of PLHIV ≥15 on ART) and from 543,005 to 1,623,925 (10–24% of PLHIV ≥15 on ART). The number of CLHIV <15 years on ART receiving MMD 3–5 months and ≥6 months increased from 66,463 to 147,180 (25–50% of CLHIV <15 on ART) and from 10,255 to 20,960 (4–7% of CLHIV <15 on ART), respectively (Figure 4).

Table 2 lists the common interventions reported by the 19 country programmes to mitigate the impact of COVID‐19 on HIV services, with specific examples from the five highlighted countries. As reflected by the quantitative data, MMD with spaced clinic visits (e.g. 6 or 12 months) were scaled up by most countries, including for children over 2 years of age. Likewise, staggered appointment schedules, social distancing in clinics, fast‐track pharmacy refills, virtual client follow‐up, and infection prevention and control (IPC) practices were scaled up by facilities and adapted by most countries. Community‐based services, such as drug pick‐up points, community‐based pharmacies and home delivery of ARVs, were scaled up to improve treatment access, especially during periods of lockdown and clinic closures. Family‐centred care models and other targeted interventions were implemented and strengthened for specific paediatric and adolescent age groups and sub‐populations. Technology‐based innovations were used to remain engaged with and support clients through short‐message systems (SMS) and telehealth. Video conferencing was used to monitor facility performance and efficiently disseminate information on COVID‐19 and its clinical management. Site‐level data dashboards and granular, multi‐level monitoring and analyses were utilized to characterize HIV cascade trends and identify any gaps to optimize client‐centred services. ARV optimization (including the scale‐up of dolutegravir‐based regimens for adults and children), commodities’ security with logistics and distribution planning, VL collection, and laboratory services and molecular capacity optimization were also reported as additional interventions to maintain PLHIV on ART with measured, durable VS.

**Table 2:** Interventions, adaptations and innovations to mitigate the impact of COVID‐19 on HIV care and treatment in Centers for Disease Control‐President's Emergency Plan for AIDS Relief‐Supported Countries

Intervention categories (19 countries)	Examples of country‐specific interventions, adaptations and innovations (five highlighted countries^a^)	Highlighted countries implementing interventions
Multi‐month ART dispensing (MMD)	Expanded MMD eligibility (e.g. MMD regardless of VL suppression status and age, MMD for TB preventive therapy)	C, D, M, N
	Pre‐packaged ARVs for fast‐track refills	C, D, M
Facility‐based client‐centred services	Expanded hours	C, D, M, N, T
	Fast‐track ART pick‐up	C, D, M, N, T
	Staggered appointments to minimize crowding	C, D, M, N, T
	Establishment of clinic adolescent hours	C, T
	Paediatric service expansion to primary healthcare facilities	C
	Establishment of hub and spoke model where high‐volume (hub) sites (repurposed to COVID‐19 centres) mentor lower‐volume (spoke) sites for ARV dispensing and client support	C, N
	Synchronized VL sample collection with ARV refills	D, M, N, T
Community‐based client service adaptations	Community ARV dispensing via mobile brigades	M, N, T
	Home ART delivery	C, M, N
	Community‐based organization and staff incentives during shutdown periods	N
	Community case management team pairing with clients who live in proximity to each other	N
	Synchronized VL sample collection with ARV refills; VLs collected in community settings	C, D, M, N
	Client feedback surveys to inform what is working or needs to be scaled	N
	Case conferencing between clinicians and community healthcare workers on clients enrolled in community programmes	N
	Culturally specific strategies for HIV screening and ARV dispensing (e.g. using chiefs’ palaces/homes as hubs)	C, N
Client tracking and tracing adaptations	Case management using an early warning dashboard (e.g. early or late missed appointments)	C, N
	Pre‐emptive interventions for treatment continuity (e.g. the use of 90‐ to 180‐day support calendars)	N
	Line‐listing and heat mapping interrupted clients’ locations and their characterization to target interventions and return to care	C, N
	Case management expansion to clients with treatment interruptions (e.g. orphans and vulnerable children [OVC] programming)	C, N
Enhanced virtual client support and education	Client telephone/SMS interactions in lieu of facility visits for psychosocial and adherence support, adverse event monitoring, defaulter tracing, test results’ delivery and education	C, D, M, N, T
	Support groups using WhatsApp and other virtual platforms to provide support without in‐person contact	N
	Phone‐based enhanced adherence counselling (EAC) for high VL clients, high VL results’ delivery and COVID‐19 messaging	C, D, M, N
	Age‐appropriate disclosure support to children and caregivers	N, T
Laboratory services optimization	Lab flow optimization to increase testing capacity and personnel training to improve lab capacity and reduce waiting times	C, D, M, N, T
	High‐throughput VL testing platform optimization	C, D, N
	VL sample movement to less saturated accredited labs for testing when central labs were repurposed for COVID‐19 testing	C, M, N
Supply chain monitoring	Commodities dashboard monitoring	C

Intervention categories (19 countries)	Examples of country‐specific interventions, adaptations and innovations (five highlighted countries^a^)	Highlighted countries implementing interventions
Technology‐based applications for planning, monitoring, communication and support	GIS technology application to geo‐map client locations and ART refill points	N
	Mobile applications to track and support private pharmacy ARV dispensing	M, N
	Video communication platforms for technical assistance, training, virtual site and partner management with daily virtual situation room meetings on HIV programme implementation	C, D, M, N, T
	COVID‐19 data dashboard and indicators to monitor site‐level intervention implementation and performance	C, M, N
	Client support using social media, SMS, toll free lines, interactive telehealth and virtual peer support	D, N
Sub‐population‐specific services	Family‐centred models with alignment of children and mother/caregiver appointments	C, D, M, N, T
	Case management services for children and adolescents, MMD and access to community‐based medication pick‐ups	C, D, M, N, T
	Mentor mother expansion to improve retention in care and VL suppression	C, D, M, N, T
	Bidirectional referrals between community OVC programmes and clinicians treating CLHIV focused on keeping families healthy, safe, stable and children with HIV in school	C, D, N, T
Infection prevention and control (IPC) practices	Revised facility flow to reduce the potential COVID‐19 exposure with triage procedures at health facility entry points	C, D, M, N, T
	Alternate work schedules for healthcare teams to maintain physical distancing while serving clients	C, D, M, N, T
	Provision of sanitizers/disinfectants and information leaflets at access points to mobilize compliance with hand hygiene	C, D, M, N, T
	COVID‐19 testing availability for patients with symptoms	C, D, M, N, T
	COVID‐19 education (e.g. hand hygiene, PPE and contact tracing)	C, D, M, N, T

Note: Initials after interventions indicate countries that reported implementing and/or scaling up intervention during the analytic period.

Abbreviations: C, Cameroon; D, DRC; M, Mozambique; N, Nigeria; T, Tanzania.

^a^ Five highlighted countries (>500,000 PLHIV and the largest net increase in the number of PLHIV on ART at the end of the analytic period, January–March 2021).

## 4 DISCUSSION

Despite the anticipated negative impact of COVID‐19 on HIV treatment services in Africa [3, 8], this analysis demonstrated continued overall growth in the total number of PLHIV on ART from the pre‐pandemic period (October 2019–December 2019) through the second pandemic wave (December 2020–March 2021) among PEPFAR‐CDC‐supported sites in 19 African countries. Despite the interruptions in observed adult HIV services between April through June 2020, just before the first wave peaked in most countries (between July and August 2020), programmes quickly recovered, leveraging and optimizing existing resources to scale up interventions that assured continued ART access, and reversing these trends by September 2020. Among the paediatric populations, however, the drop in ART initiations did not return to pre‐pandemic levels and there was limited growth in the number of children on ART over the analytic period, indicating limited progress with paediatric HIV treatment programmes during this period. While both VLC and suppression improved for both adults and children, challenges remain to achieve higher rates of VLC and suppression for all PLHIV on ART. The second wave of COVID‐19 appeared to have less impact on adult treatment outcomes than the first wave, suggesting the interventions and adaptations implemented by countries may have helped to mitigate the impact of COVID‐19 and support HIV treatment programme performance recovery. Our overall analysis findings suggest the MOH‐led, PEPFAR‐CDC‐supported programmes in these 19 countries were robust, resilient programmes that were capable of quickly adopting and adapting service delivery models and interventions to meet the needs of clients.

The key interventions that HIV programmes implemented aligned with the priorities of treatment continuity, IPC, leveraging health systems and flexible person‐centred service delivery. Further, many governments quickly adopted policies that facilitated the implementation of certain interventions [27]. For example, patient eligibility requirements for MMD were relaxed such that a larger number of PLHIV, including children, became eligible regardless of their VL or length of time on ART [28]. Implementation and scale‐up of interventions that reduced visit frequency made facilities safer from COVID‐19 transmission and brought treatment services to communities. The use of technology and telehealth were among the most common innovative interventions to engage, support and educate PLHIV, and virtual platforms were used for rapid information dissemination to providers and to conduct virtual site‐level monitoring for gap identification and remediation [29, 30].

Based on a combined analysis of the data from 19 countries, the decline in new ART initiations between April through June 2020 was likely due in part to the decline in HIV testing that occurred during this same time period. HIV testing was shown to decrease across all modalities from January to March and April to June 2020 by 24.6–30.2% in 14 PEPFAR‐supported countries. However, HIV testing increased across all modalities by July–September 2020 (range 26.1–65.1%) showing a similar pattern as ART initiations [31]. While improvements in treatment interruptions were also observed for children and adolescents, declining to even lower than pre‐pandemic rates by March 2021, limited recovery in new ART initiations, without reaching pre‐pandemic levels was evident. Analyses of the treatment trends in five highlighted countries demonstrated that large net increases in the number of adults currently on ART could be attributed either to a remarkable rebound in new ART initiations, with steep increases to above pre‐pandemic levels (four countries), or substantial success in steadily reducing treatment interruptions to below pre‐pandemic levels by March 2021 (one country). Among children, while four of the five countries were able to reduce treatment interruptions to pre‐pandemic levels or below, new ART initiations did not see the steep increases in adults, likely explaining the minimal to no growth in the net number of children currently on ART. Similar to the trends of the 19 countries combined, among both adults and children in the five highlighted countries, a higher proportion of PLHIV on ART for <3 months versus PLHIV on ART ≥ 3 months experienced treatment interruptions, underscoring the need for targeted interventions to support early retention on ART. Such support may include regular and frequent contact with healthcare staff or peer counsellors, adherence counselling and support, and treatment access/pick‐up options outside facilities.

Factors contributing to the challenges observed in paediatric programme performance need further investigation and urgent attention. Potential factors may include caregivers’ reluctance to bring their children to facilities due to fear of COVID‐19 exposure or to community‐based services due to stigma and fear of disclosure, children ageing out of the paediatric programme, disruptions due to clinic and school closures, reductions in paediatric case finding and reductions in maternal to child HIV transmission [32]. Although the implementation of family‐centred models of care [33] was reported by countries, the degree of scale‐up during this period was not measured. Among adolescents, the suspension of peer support groups may have also had an impact on retention [34]. While a chronic challenge for paediatric and adolescent populations, the trends observed in paediatric treatment interruptions suggest that age‐specific and targeted interventions, including the continued expansion of MMD, access to community‐based refills and case management services, may have improved IIT [35].

This study has several limitations. Routine programme data are reported as quarterly cross‐sectional aggregated results and have been analysed at the country level. Associations with reported site‐level programme interventions implemented to mitigate the impact of COVID‐19 cannot be directly correlated to specific patients or site outcomes. We, therefore, could not establish direct causality, effectiveness hierarchy or timing impact of the summarized interventions with the observed treatment trends given the observational nature of this analysis. However, coinciding with the implementation of these multiple interventions, we observed an improvement in new treatment initiations and a decline in treatment interruptions between the first and second pandemic waves. Sites excluded from analyses may have been more impacted by periodic COVID‐19‐related disruptions or other reasons, such as sites no longer receiving PEPFAR support or reassignment to another agency other than CDC for support and may have impacted estimates of treatment initiations and interruptions. Although we assessed IIT occurring in a specific quarter, aggregated site‐level data may not necessarily reflect the final outcome (e.g. death, transfer, etc.), and we were not able to evaluate successes in returning persons to treatment, as the indicator for this changed in October 2020, making interpretation for the entire analytic period difficult. Limited national patient unique identification systems may have led to “new ART initiations” of individuals who transferred from another clinic without reporting previous care or treatment (silent transfer) or returned to care/ART after an interruption. Misclassification of reported treatment interruptions has been characterized in multiple studies, although recent reports have observed this to be decreasing [36]. A recent article from six Southern African countries found that 17% of clients who were lost to follow‐up were categorized as silent transfers, 11% were dead and 12% were misclassified as “lost” [37]. While silent transfers, deaths and patients wrongly categorized as “lost” may have been affected by COVID‐19‐related disruptions in the current study population, given the analysis variable used was IIT, these clients reflect a consistent bias that would have been included in interruptions reported across the time period. VLC was not reported in the period between October 2019 and March 2020 due to not having the data for PLHIV eligible for VL monitoring, potentially failing to capture VL trend dynamics in the early period when COVID‐19 restrictive measures were implemented. Data quality assurance occurs from checks at multiple levels during the PEPFAR reporting process; however, issues with programme data completeness and indicator disaggregation during the analytic period across sites could not be excluded. Finally, an expanded exploration of the programme interventions implemented to mitigate the impact of COVID‐19 in the countries was limited to the five countries presented and does not reflect a comprehensive picture of all countries’ innovations and adaptations in HIV treatment services implemented and inform future programming.

## 5 CONCLUSIONS

This study showed that while COVID‐19 impacted HIV treatment services across 19 African countries, rapid recovery was observed overall in adult populations and coincided with the reported implementation and scale‐up of innovative interventions in health facilities and communities. Overall, children on ART were left behind during the COVID‐19 period analysed, highlighting the need for population‐specific interventions to ensure that CLHIV are identified, maintain access and remain on treatment. The lessons learned from innovations in HIV treatment service delivery thus far in the COVID‐19 pandemic will likely better position African countries to ensure the gains in HIV programmes can be protected and accelerated despite the unforeseen threats that may lie ahead.

## COMPETING INTERESTS

The authors report no competing interests.

## AUTHORS’ CONTRIBUTIONS

All authors have read and approved the final draft of this manuscript. PJB, HMC, NM, JAG, KAP, MWS, SL, CZ, TE and KM conceptualized and developed the methodology for the paper and wrote the first draft. NM, MWS and JAG conducted the data analyses. PJB, HMC, NM, JAG, KAP, MWS, SL, CZ, DKM, MLK, EKD, LB, SB, EE, PF, AMN, SM, VNO, FT, MG, EMK, AM, FMA, TFT, AFA, RN, APDL, IG, SYH, LA, CO, AI, CM, EY, SM, ZP, SB, JR, DJM, BM, LJN, CK, SA, LBM, PN, OM, TE and KM contributed to the content, review and editing of the manuscript.

## FUNDING

This publication has been supported by the President's Emergency Plan for AIDS Relief (PEPFAR) through the Centers for Disease Control and Prevention (CDC).

## DISCLAIMER

The findings and conclusions in this report are those of the authors and do not necessarily represent the official position of the funding agencies.

## Data Availability

The data that support the findings of this study are available on request from the corresponding author. The data are not publicly available due to privacy or ethical restrictions.

## References

[1] World Health Organization . WHO COVID19_Cases (arcgis.com). Geneva: WHO; 2021.

[2] El‐Sadr WM , Justman J . Africa in the path of COVID‐19. N Engl J Med. 2020;383:e11. 32302075 10.1056/NEJMp2008193

[3] Nuwagira E , Muzoora C . Is sub‐Saharan Africa prepared for COVID‐19? Trop Med Health. 2020;48:18. 32288543 10.1186/s41182-020-00206-x PMC7146014

[4] Geretti AM , Stockdale AJ , Kelly SH , Cevik M , Collins S , Waters L , et al. Outcomes of coronavirus disease 2019 (COVID‐19) related hospitalization among people with human immunodeficiency virus (HIV) in the ISARIC World Health Organization (WHO) Clinical Characterization Protocol (UK): a prospective observational study. Clin Infect Dis. 2021;73(7):e2095–106. 33095853 10.1093/cid/ciaa1605 PMC7665382

[5] Barbera LK , Kamis KF , Rowan SE , Davis AJ , Shehata S , Carlson JJ , et al. HIV and COVID‐19: review of clinical course and outcomes. HIV Res Clin Pract. 2021;22(4):102–18. 34514963 10.1080/25787489.2021.1975608 PMC8442751

[6] Nagarakanti SR , Okoh AK , Grinberg S , Bishburg E . Clinical outcomes of patients with COVID‐19 and HIV coinfection. J Med Virol. 2021;93(3):1687–93. 32949148 10.1002/jmv.26533 PMC7537324

[7] UNAIDS . Global HIV & AIDS statistics — fact sheet. Geneva: UNAIDS; 2021.

[8] Jewell BL , Mudimu E , Stover J , ten Brink D , Phillips AN , Smith JA , et al. Potential effects of disruption to HIV programmes in sub‐Saharan Africa caused by COVID‐19: results from multiple mathematical models. Lancet HIV. 2020;7(9):e629–40. 32771089 10.1016/S2352-3018(20)30211-3 PMC7482434

[9] UNAIDS . Fast track: fast‐track strategy to end the AIDS epidemic by 2030. Geneva: UNAIDS; 2014.

[10] The President's Emergency Plan for AIDS Relief (PEPFAR) . About us – PEPFAR. Washington, DC: United States Department of State; 2021.

[11] Prust ML , Banda CK , Nyirenda R , Chimbwandira F , Kalua T , Jahn A , et al. Multi‐month prescriptions, fast‐track refills, and community ART groups: results from a process evaluation in Malawi on using differentiated models of care to achieve national HIV treatment goals. J Int AIDS Soc. 2017;20(Suppl 4):41–50. 10.7448/IAS.20.5.21650 PMC5577715 28770594

[12] Roy M , Bolton Moore C , Sikazwe I , Holmes CB . A review of differentiated service delivery for HIV treatment: effectiveness, mechanisms, targeting, and scale. Curr HIV/AIDS Rep. 2019;16:324–34. 31230342 10.1007/s11904-019-00454-5

[13] World Health Organization . Consolidated guidelines on the use of antiretroviral drugs for treating and preventing HIV infection: recommendations for a public health approach. 2nd ed. Geneva: WHO; 2016. 27466667

[14] Jobarteh K , Shiraishi RW , Malimane I , Samo Gudo P , Decroo T , Auld AF , et al. Community ART support groups in Mozambique: the potential of patients as partners in care. PLoS One. 2016;11(12):e0166444. 27907084 10.1371/journal.pone.0166444 PMC5132187

[15] Penn AW , Azman H , Horvath H , Taylor KD , Hickey MD , Rajan J , et al. Supportive interventions to improve retention on ART in people with HIV in low‐ and middle‐income countries: a systematic review. PLoS One. 2018;13(12):e0208814. 30550574 10.1371/journal.pone.0208814 PMC6294385

[16] Long L , Kuchukhidze S , Pascoe S , Nichols BE , Fox MP , Cele R , et al. Retention in care and viral suppression in differentiated service delivery models for HIV treatment delivery in sub‐Saharan Africa: a rapid systematic review. J Int AIDS Soc. 2020;23(11):e25640 33247517 10.1002/jia2.25640 PMC7696000

[17] Hoffman RM , Moyo C , Balakasi K , Siwale Z , Hubbard J , Bardon A , et al. Multimonth dispensing of up to 6 months of antiretroviral therapy in Malawi and Zambia (INTERVAL): a cluster randomised, non‐blinded, non‐inferiority trial. Lancet. 2021;9:e628–38. 10.1016/S2214-109X(21)00039-5 33865471

[18] Wringe A , Cawlely C , Szumilin E , Salumu L , Amoros Quiles I , Pasquier E , et al. Retention in care among clinically stable antiretroviral therapy patients following a six‐monthly clinical consultation schedule: findings from a cohort study in rural Malawi. J Int AIDS Soc. 2018;21:e25207. 30450699 10.1002/jia2.25207 PMC6240757

[19] Tukei BB , Fatti G , Tiam A , Ngorima‐Mabhena N , Tukei VJ , Tshabalala I , et al. Twelve‐month outcomes of community‐based differentiated models of multimonth dispensing of ART among stable HIV‐infected adults in Lesotho: a cluster‐randomized noninferiority trial. J Acquir Immune Defic Syndr. 2020;85(3):280–91. 32665460 10.1097/QAI.0000000000002439

[20] World Health Organization . Disruption in HIV, hepatitis and STI services due to COVID‐19. Geneva: WHO; 2020.

[21] The Global Fund . The impact of COVID‐19 on HIV, TB and malaria services and systems for health: a snapshot from 502 health facilities across Africa and Asia. Geneva: 2021.

[22] U.S. State Department . The President's Emergency Plan for AIDS Relief (PEPFAR). Washington, DC. Accessed on September 8, 2021. Available from: 07.01.2020-PEPFAR-Technical-Guidance-During-COVID.pdf (state.gov).

[23] Wilkinson L , Grimsrud A . The time is now: expedited HIV differentiated service delivery during the COVID‐19 pandemic. J Int AIDS Soc. 2020;23(5):e25503. 32378345 10.1002/jia2.25503 PMC7203569

[24] Golin R , Godfrey C , Firth J , Lee L , Minior T , Phelps BR , et al. PEPFAR's response to the convergence of the HIV and COVID‐19 pandemics in sub‐Saharan Africa. J Int AIDS Soc. 2020;23(8):e25587. 32767707 10.1002/jia2.25587 PMC7405155

[25] DATIM (Data for Accountability, Transparency and Impact) . DATIM (zendesk.com). PEPFAR, accessed October 13, 2021.

[26] US Department of State. Monitoring, Evaluation, and Reporting Indicator Reference Guide . MER 2.0 (Version 2.5). FY21 MER 2.5 Indicator Reference Guide (state.gov). September 2020.

[27] Grimsrud A , Wilkinson L . Acceleration of differentiated service delivery for HIV treatment in sub‐Saharan Africa during COVID‐19. J Int AIDS Soc. 2021;24:e25704. 34105884 10.1002/jia2.25704 PMC8188395

[28] Bailey L , Siberry G , Agaba P , Douglas M , Clinkscales J , Godfrey C . The impact of COVID‐19 on multi‐month dispensing (MMD) policies on antiretroviral therapy (ART) and MMD uptake in 21 PEPFAR‐supported countries: a multi‐country analysis. J Int AIDS Soc. 2021;24(6). 10.1002/jia2.25794 PMC8554217 34713578

[29] Feroz AS , Khoja A , Saleem S . Equipping community health workers with digital tools for pandemic response in LMICs. Arch Public Health. 2021;79(1):1. 33390163 10.1186/s13690-020-00513-z PMC7779158

[30] Talisuna AO , Bonkoungou B , Mosha FS , Struminger BB , Lehmer J , Arora S , et al. The COVID‐19 pandemic: broad partnerships for the rapid scale up of innovative virtual approaches for capacity building and credible information dissemination in Africa. Pan Afr Med J. 2020;37:255. 33598070 10.11604/pamj.2020.37.255.23787 PMC7864260

[31] Drammeh B , Dee A , Lasry A , Medley A , Aholou T , Yee R , Dale H , et al. Changes in HIV testing services after COVID‐19 in 11 sub‐Saharan Africa countries. Conference on Retroviruses and Opportunistic Infections (CROI), Virtual 2021, Oral presentation of abstract 143.

[32] Vrazo AC , Golin R , Fernando NB , Killam WP , Sharifi S , Phelps BR , et al. Adapting HIV services for pregnant and breastfeeding women, infants, children, adolescents and families in resource‐constrained settings during the COVID‐19 pandemic. J Int AIDS Soc. 2020;23(9):e25622. 32996705 10.1002/jia2.25622 PMC7525801

[33] Wilkinson L , Siberry GK , Golin R , Phelps BR , Wolf HT , Modi S , et al. Children and their families are entitled to the benefits of differentiated ART delivery. J Int AIDS Soc. 2020;23(4):e25482. 32239657 10.1002/jia2.25482 PMC7113528

[34] Mark D , Hrapcak S , Ameyan W , Lovich R , Ronan A , Schmitz K , et al. Peer support for adolescents and young people living with HIV in sub‐Saharan Africa: emerging insights and a methodological agenda. Curr HIV/AIDS Rep. 2019;16(6):467–74. 31776974 10.1007/s11904-019-00470-5 PMC6945499

[35] Enane LA , Davies MA , Leroy V , Edmonds A , Apondi E , Adedimeji A , et al. Traversing the cascade: urgent research priorities for implementing the ‘treat all’ strategy for children and adolescents living with HIV in sub‐Saharan Africa. J Virus Erad. 2018;4(2):40–6. 30515313 10.1016/S2055-6640(20)30344-7 PMC6248846

[36] Zurcher K , Mooser A , Anderegg N , Tymejckzyk O , Couvillon M , Nash D , et al. Outcomes of HIV‐positive patients lost to follow‐up in African treatment programmes. Trop Med Int Health. 2017;22(4):375–87. 28102610 10.1111/tmi.12843 PMC5580236

[37] Ballif M , Christ B , Anderegg N , Chammartin F , Muhairwe J , Jefferys L , et al. Tracing people living with human immunodeficiency virus who are lost to follow‐up at antiretroviral therapy programs in Southern Africa: a sampling‐based cohort study in 6 countries. Clin Infect Dis. 2022;74(2):171–9. 33993219 10.1093/cid/ciab428 PMC8800181

